# Resolvin D1 Improves the Treg/Th17 Imbalance in Systemic Lupus Erythematosus Through miR-30e-5p

**DOI:** 10.3389/fimmu.2021.668760

**Published:** 2021-05-19

**Authors:** Tao Cheng, Shuai Ding, Shanshan Liu, Xiaojing Li, Xiaojun Tang, Lingyun Sun

**Affiliations:** Department of Rheumatology and Immunology, The Affiliated Nanjing Drum Tower Hospital, Medical School of Nanjing University, Nanjing, China

**Keywords:** Resolvin D1, systemic lupus erythematosus, regulatory T cells, T helper 17 cells, miR-30e-5p

## Abstract

Resolvin D1 (RvD1) prompts inflammation resolution and regulates immune responses. We explored the effect of RvD1 on systemic lupus erythematosus (SLE) and investigated the correlation between RvD1 and Treg/Th17 imbalance, which is one of the major factors contributing to the pathogenesis of disease. SLE patients and healthy controls were recruited to determine plasma RvD1 levels. MRL/*lpr* lupus model was used to verify rescue of the disease phenotype along with Treg/Th17 ratio. Purified naive CD4+ T cells were used to study the effect of RvD1 on Treg/Th17 differentiation *in vitro*. Furthermore, small RNA Sequencing and transfection were performed successively to investigate downstream microRNAs. The result showed that the RvD1 level was significantly lower in active SLE patients compared with inactive status and controls. Moreover, The SLE disease activity index (SLEDAI) score had a significant negative correlation with RvD1 level. As expected, RvD1 treatment ameliorated disease phenotype and inflammatory response, improved the imbalanced Treg/Th17 in MRL/*lpr* mice. In addition, RvD1 increased Treg while reduced Th17 differentiation *in vitro*. Furthermore, miR-30e-5p was verified to modulate the Treg/Th17 differentiation from naïve CD4+ T cells as RvD1 downstream microRNA. In conclusion, RvD1 effectively ameliorates SLE progression through up-regulating Treg and down-regulating Th17 cells *via* miR-30e-5p.

## Introduction

Systemic lupus erythematosus (SLE) is a systemic autoimmune disease, which could be a consequence of failure to resolve inflammation and restore T cell homeostasis. Accumulating evidences suggested that the imbalance of regulatory T-cells (Treg) and T-helper 17 (Th17) contribute to the pathogenesis of SLE (1, 2). Circulating Treg decrease during disease flares in SLE patients, which mediate the anti-inflammatory response by secreting IL-10 and transforming growth factor (TGF)-β and maintain the state of autoimmune tolerance (3). In addition, the number of Th17 cells, as well as Th17-related inflammatory cytokines such as interleukin 17 (IL-17), IL-6, were found increased in SLE patients (4). Previous evidences showed that oxidative stress and STAT signaling pathways contribute to the Treg/Th17 dysfunction in the pathogenesis of SLE (5, 6). The underlying reasons for Treg/Th17 imbalance in SLE are not clear yet.

Several recent studies have demonstrated that the specialized pro-resolving lipid mediators (SPM), a superfamily of pro-resolving lipids that derive metabolically from ω-3 and ω-6 essential fatty acids, play an important role in the chronic autoimmune and inflammatory diseases (7). Resolvin D1 (RvD1) is one of the identified mediators, which is produced from docosahexaenoic acid. RvD1 inhibits polymorphonuclear neutrophils penetration and thereby regulating its activity to enhance phagocytosis and ultimately leading to apoptotic cells and bacteria clearance in both *vivo* and *vitro* environments in acute inflammation (7, 8). The effects of RvD1 on chronic inflammatory disease have also attracted attention in recent years. Lima-Garcia et al. discovered that RvD1 could relieve pain in adjuvant-induced arthritis in rats (9). Sun et al. reported that RvD1 could suppress pannus formation in Rheumatoid Arthritis (RA) (10). A preliminary study has reported the first evidence of a potential association between SLE and RvD1 plasma level (11). To demonstrate the effect of RvD1 on Treg/Th17 balance, we performed RvD1 tests in hospital recruited SLE patients, couple with induction of Treg and Th17 in the presence of RvD1 *in vitro*.

RvD1 exerts pro-resolving effects through G protein–coupled receptors (GPCRs), and besides, microRNAs (miRNAs) are part of the mechanisms of RvD1 actions (12). MiRNAs, a class of single-stranded small non-coding RNAs with 19-23 nucleotides in length, could serve as critical regulators in resolution circuits. For example, RvD1 regulates miR-219 and miR-208a endogenously expressed in resident peritoneal cells from human GPCRs overexpressing transgenic mice (13). This enlightens us to identify the prominently regulated miRNAs and reveal the mechanism involving RvD1 actions in SLE.

In this study, we investigated the protective effect of RvD1 on SLE and Treg differentiation, and further elucidated the microRNA-mediated regulation involved in controlling of Treg/Th17 balance.

## Materials and Methods

### Patients

Totally, 35 SLE patients and 8 healthy subjects were included in this study at the Affiliated Drum Tower Hospital, Medical School of Nanjing University. The average age of the SLE patients and healthy controls were 33.5 ± 9.6 and 35.6 ± 10.0, respectively, and the gender ratio (female/male) of the SLE patients and healthy controls were 22/4 and 6/2, respectively. The two groups were age- and gender- matched. All the subjects were given informed consents for the collection of peripheral blood. All SLE patients fulfilled the 1997 revised criteria of the American College of Rheumatology for SLE (14). The clinical details of the patients are shown in online **Supplementary Table 1**. Patients were excluded from the study if they had the following conditions: uncontrolled infection including pneumonia (bacterial, virus, or fungal), pulmonary tuberculosis, hepatitis B and C, skin infection, central nervous system infection; persistent or unresolved inflammation including atherosclerosis, chronic obstructive pulmonary disease, diabetes, rheumatoid arthritis, Alzheimer’s and amyotrophic lateral sclerosis; patient who has taken aspirin or sea fish oil within one month; cancer at enrollment or in the previous 5 years; woman who was pregnant or lactating. According to the SLEDAI-2000, the disease activity of SLE patients was scored, SLEDAI score<10 was considered as inactive condition, while SLEDAI score≥10 was considered as active condition. The study was approved by the Ethics Committee of The Affiliated Nanjing Drum Tower Hospital, Medical School of Nanjing University.

### Mice

Female MRL/*lpr* mice were purchased from Shanghai SLAC Laboratory Animal Co. Ltd. (Shanghai, China). Mice were housed under specific-pathogen-free conditions in the animal center of the Affiliated Drum Tower Hospital. All experimental animal protocols were approved by the Committee of Experimental Animal Administration of The Affiliated Nanjing Drum Tower Hospital, Medical School of Nanjing University.

### Histopathology Examination

Kidneys obtained at sacrifice were divided along their longitudinal axis, which were fixed in 10% formaldehyde and embedded in paraffin. Three-micrometer-thick sections were stained with hematoxylin-eosin (HE) for histopathology. Sections were photographed using a fluorescence microscope fitted with a digital camera (Cannon Power shot G10, Cannon, Inc.). Histological scores of renal lesions were calculated. Mainly, the severity of glomerulonephritis was graded on a 0-4 scale as follows: 0, normal; 1, mild increase in mesangial cellularity and matrix; 2, moderate increase in mesangial cellularity and matrix, with thickening of the glomerular basement membrane (GBM); 3, focal endocapillary hypercellularity with obliteration of capillary lumina and a substantial increase in the thickness and irregularity of the GBM; and 4, diffuse endocapillary hypercellularity, segmental necrosis, crescents and hyalinized end-stage glomeruli (15).

### Enzyme-Linked Immunosorbent Assay (ELISA)

RvD1 plasma level was measured by ELISA according to the manufacturer’s protocol (Cayman Chemical Co., Ann Arbor, MI). Plasma levels of ANA, anti-dsDNA antibody, and total IgG, TNF-α, interferon (IFN)-γ, IL-6, IL-10, IL-17 and TGF-β were measured using a Mouse ANA ELISA Kit (Alpha Diagnostic, USA), a Mouse anti-dsDNA ELISA Kit (Shibayagi, Japan), and Mouse IgG total, TNF-α, IFN-γ, IL-6, IL-10, IL-17, TGF-β ELISA Kits (Fcmacs, China) respectively, according to the manufacturer’s instructions.

### Flow Cytometry

Spleen and lymph nodes from MRL/*lpr* mice were gently triturated with a 300-mesh cell strainer. The strained cells were washed twice with phosphate buffered saline and suspended in RPMI 1640 medium.

For the assessment of Th17 cells, lymphocytes were stimulated for 4 h with phorbol myristate acetate (50 ng/ml), Ionomycin (1 µg/ml) and brefeldin A (5 µg/ml). Cells were then stained with APC conjugated anti-CD4 for 30 min in the dark at 4°C. Subsequently, the cells were fixed and permeabilized using Cytofix/Cytoperm for 30 min in the dark at 4°C. The cells were then washed in 0.05% saponin before being labeled intracellularly with PE conjugated anti-IL-17A for 1 h in the dark at 4°C. Similarly, for the assessment of Treg cells, cells were stained with FITC conjugated anti-CD4 and APC conjugated anti-CD25. Following fixing and permeabilizing, cells were washed before being stained with PE-conjugated anti-FoxP3 intracellularly. Lymphocytes were then washed in 0.05% saponin, resuspended in a 300-µl flow cytometry staining buffer and then analyzed using a FACS Calibur flow cytometer. The cytometric data were analyzed with FlowJo software. All the reagents involved in flow cytometry were purchased from BD Biosciences.

### Quantitative Real-Time Polymerase Chain Reaction (RT-PCR) Analysis

Total cellular RNA was extracted using Trizol reagent (Invitrogen) and 200 ng RNA was used in RT reactions. MiRNAs were performed by poly(A)-tailed RT-PCR as previously described (16). MiR-30e-5p primers and the 3′primer were: 5′-UGUAAACAUCCUUGACUGGAAG-3′; 5′-ATTCTAGAGGCCGAGGCGGCCGACATGT-3′. Sequences of the U6 forward and reverse primers were: 5′-CGCTTCGGCAGCACATATACTA-3′; 5′-CGCTTCACGAATTTGCGTGTC-3′. For real-time PCR experiments, reactions containing SYBR Premix EX Taq (Takara), ROX Reference Dye (50; Takara), cDNA, and gene primers were run on a Step One Plus real-time PCR system and analyzed using Step One Software, version 2.1 (Applied Biosystems). Relative gene quantification was calculated by the 2ΔCt method and then normalized to the level of U6.

### Naive CD4+ T Cell Differentiation and Transfection *In Vitro*

The subpopulation of CD4+ T lymphocytes was purified by immunomagnetic negative isolation using the EasySep™ human naïve CD4+ T Cell Isolation Kit II (Stem cell, Canadian). The naïve CD4+ T cells were seeded at density of 3×10^5^ cells/well in a 96-well plate. The cells were then transfected either with mock control or with miR-30e-5p mimic or inhibitor obtained from RiboBio (Guangzhou, China) using Entranster™-R4000 Transfection reagent from Engreen (Beijing, China). The cells were then harvested and activated by plate-bound anti-CD3 (2 μg/ml) and anti-CD28 (2 μg/ml) antibodies, with the addition of recombinant human TGF-β (5 ng/ml) and IL-2 (5 ng/ml) to induce Treg cell conversion or human IL4 (10 μg/ml), IFN-γ (10 μg/ml), TGF-β (2.5 ng/ml), IL-6 (10 ng/ml), and IL-23 (10 ng/ml) to induce Th17 cell conversion. In some cultures, RvD1 (10 nM) was added once a day for 4 days. Cells were collected for measurement of Treg or Th17 cells on the fifth day.

### UMI Small RNA Sequencing and Data Analysis

Total RNA containing small RNA was extracted from naive CD4+ T-cells using a mirVana miRNA Isolation Kit (Ambion, Austin, TX, USA). RNA degradation and contamination was monitored on 1% agarose gels. RNA purity was checked using the NanoPhotometer^®^ spectrophotometer (IMPLEN, CA, USA). RNA concentration was measured using Qubit^®^ RNA Assay Kit in Qubit^®^ 2.0 Flurometer (Life Technologies, CA, USA). RNA integrity was assessed using the RNA Nano 6000 Assay Kit of the Agilent Bioanalyzer 2100 system (Agilent Technologies, CA, USA). A total amount of 200 ng total RNA per sample was used as input material for the small RNA library. Sequencing libraries were generated using QIAseq miRNA Lib kit following manufacturer’s recommendations and index codes were added to attribute sequences to each sample. Briefly, A pre-adenylated DNA adapter was directly and specifically ligated to 3′ end of miRNA, siRNA, and piRNA. After the 3′ ligation reaction, an RNA adapter is ligated to the 5′ends. The RT primer containing an integrated UMI subsequently binds to a region of the 3′ adapter and facilitates conversion of the ligated molecules into cDNA while assigning a UMI to every miRNA. During reverse transcription, a universal sequence is also added that is recognized by the sample indexing primers. After reverse transcription, a cleanup of the cDNA is performed using a streamlined magnetic bead-based method. PCR amplification was performed using the universal forward and reverse primer-containing index. Afterwards, a cleanup of the library is performed using a streamlined magnetic bead-based method. At last, library quality was assessed on the Agilent Bioanalyzer 2100 system using DNA High Sensitivity Chips. The clustering of the index-coded samples was performed on a cBot Cluster Generation System using TruSeq SR Cluster Kit v3-cBot-HS (Illumia) according to the manufacturer’s instructions. After cluster generation, the library preparations were sequenced on an Illumina platform and 150 bp paired-end reads were generated.

Raw reads of fastq were firstly processed through custom perl and python cripyts. Clean data (clean reads) were obtained by cutting the head 100 bases from each raw read and removing low quality reads from them. At the same time, Q20, Q30, and GC-content of the clean data were calculated. Then, a certain range of length from clean reads that have correct UMI pattern were extracted by UMI-tools v1.0.0. All the downstream analyses were based on the clean UMI reads with high quality. Mapping reads to the reference sequence by Bowtie and deduplicating reads by UMI reads mapping coordinates were processed successively. Mapped small RNA tags were used to looking for known miRNA. MiRBase20.0 was used as reference, modified software mirdeep2 and srna-tools-cli were used to obtain the potential miRNA and draw the secondary structures. Custom scripts were used to obtain the miRNA counts as well as base bias on the first position of identified miRNA with certain length and on each position of all identified miRNA respectively. Tags originating from protein-coding genes were removed by mapping to RepeatMasker, Rfam database. The software miREvo and mirdeep2 were integrated to predict novel miRNA. At the same time, custom scripts were used to obtain the identified miRNA counts as well as base bias on the first position with certain length and on each position of all identified miRNA respectively. Summarizing all alignments and annotations obtained before. The total rRNA proportion was used a marker as sample quality indicator. It should be less than 40% as high quality. In our analysis pipeline, miRNA, which might have base edit could be detected by aligning all the sRNA tags to mature miRNA, allowing one mismatch. Known miRNA used miFam.dat (http://www.mirbase.org/ftp.shtml) to look for families; novel miRNA precursor was submitted to Rfam (http://rfam.sanger.ac.uk/search/) to look for Rfam families. Next, predicting the target gene of miRNA was performed by psRobot_tar in miRanda. miRNA expression levels were estimated by TPM (transcript per million) through the following criteria: Normalization formula: Normalized expression=mapped readcount/Total reads*1000000. Differential expression analysis of two samples was performed using the DEGseq (2010) R package. P-value was adjusted using qvalue. qvalue<0.01 and |log2 (foldchange)|>1 was set as the threshold for significantly differential expression by default. Gene Ontology (GO) enrichment analysis was used on the target gene candidates of differentially expressed miRNAs. GOseq based Wallenius non-central hyper-geometric distribution was implemented for GO enrichment analysis. We used KOBAS software to test the statistical enrichment of the target gene candidates in KEGG pathways.

### Statistical Analysis

The data were expressed as mean ± SEM and analyzed with Prism 5 (GraphPad Software). Student’s t test was used to analyze significance between two groups and comparisons between more than two groups were analyzed using one-way ANOVA. The value of *p*<0.05 was considered statistically significant.

## Results

### RvD1 Level Decreased in Patients With Active SLE

**Figure 1:**
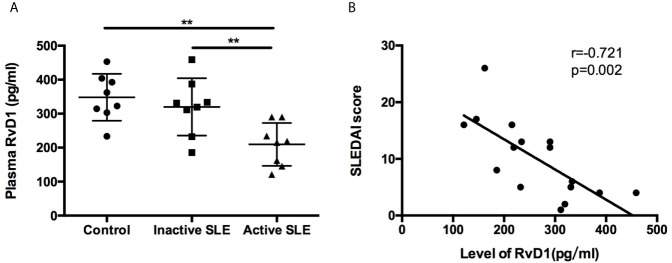
Plasma RvD1 level in SLE patients. **(A)** The levels of RvD1 in plasma samples from healthy controls, patients with inactive and active SLE (n=8 per group). **(B)** The SLEDAI score had a significant negative correlation with RvD1 level (r=-0.721, p=0.002). All data are mean ± SEM. **p < 0.01.

The plasma RvD1 level was significantly lower in active SLE patients compared with control subjects (**Figure 1A**). However, no significant difference was observed between control and inactive SLE patients. We used the SLEDAI score to explore the relationship between disease activity of SLE and the RvD1 level. Negative correlation of the RvD1 level and the SLEDAI score was observed with r=−0.721, p=0.002 (**Figure 1B**). However, There is no correlation between RvD1 level and IgG, complement, erythrocyte sedimentation rate, ANA level or 24-h urine protein quantification. RvD1 level and dsDNA antibody level had a correlation trend in 10 patients, r=-0.629, p=0.051 (**Supplementary Figure 1**).

### RvD1 Ameliorated Disease in MRL/*lpr* Mice

**Figure 2:**
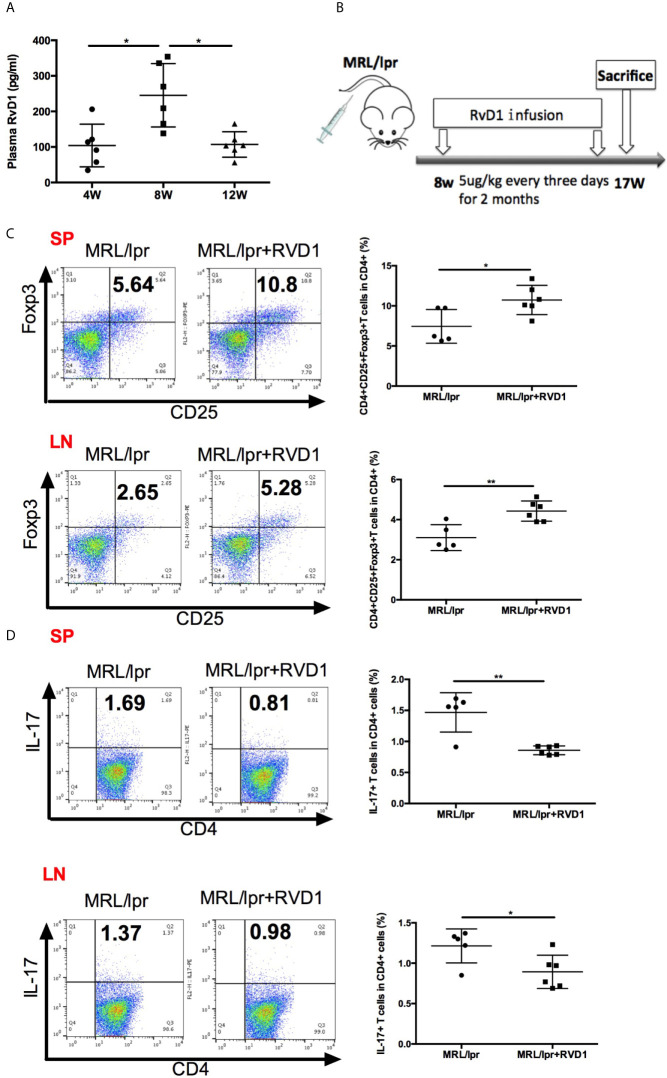
RvD1 influence the Treg/Th17 balance in MRL/*lpr* mice. **(A)** Quantification with ELISA of RvD1 in the plasma of MRL/*lpr* mice at 4, 8 and 12 weeks of age. **(B)** RvD1 treatment scheme (5μg/kg) injections were performed every three days for 2 months, starting from 8 weeks of age. **(C, D)** Representative FACS analysis of Treg and Th17 of spleens and cervical lymph nodes. All data are mean ± SEM. n=5 in MRL/*lpr* group; n=6 in MRL/*lp*r+RvD1 treated group. *p < 0.05; **p < 0.01.

In the early stages of self-limited acute inflammation, SPM have been depicted on the time lines and recognized to play an important role (17). We questioned whether MRL/*lpr* mice model could represent the similar alteration in the resolution of inflammation, based on the assessment of plasma RvD1 level. Interestingly, 8-week-old MRL/*lpr* mice showed higher plasma level of RvD1 (**Figure 2A**). Time-course changes of plasma RvD1 concentration suggest that 8-week-old MRL/*lpr* mice receiving RvD1 boost could help relieve inflammation in its initial stages.

We next assessed the effects of RvD1 on MRL*/lpr* mice *in vivo.* 8-week-old MRL*/lpr* mice were injected with 5ug/kg RvD1 or saline every 3 days for 2 months (**Figure 2B**). 48 h after the last injection splenic cells and cervical lymph nodes cells were analyzed by flow cytometry to evaluate the Treg/Th17 percentage. We found that Treg cells were significantly increased and Th17 cells were markedly reduced in both spleens and cervical lymph nodes T-lymphocytes from RvD1-treated mice (**Figures 2C, D**).

**Figure 3:**
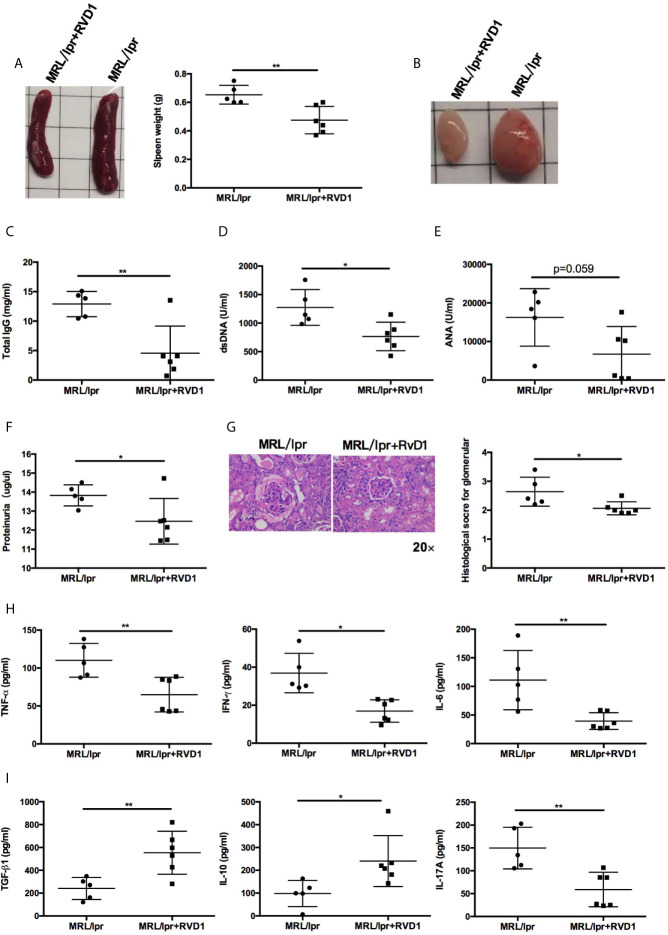
RvD1 treatment ameliorates disease phenotype and inflammatory response in MRL/*lpr* mice. RvD1 treatment reduced spleen index **(A)**, lymph node size **(B)**, plasma levels of total IgG **(C)**, anti-dsDNA antibodies **(D)**, ANA **(E)**, proteinuria **(F)**, glomerular enlargement and hypercellularity **(G)**, pro-inflammatory cytokines (TNF-α, IFN-γ and IL-6) **(H)** and a Th17 cell-related cytokine (IL-17) **(I)**, while increase Treg cell-related cytokines (IL-10 and TGF-β) **(I)** in MRL/*lpr* mice. All data are mean ± SEM. n=5 in MRL/*lpr* group; n=6 in MRL/*lp*r+RvD1 treated group. *p < 0.05; **p < 0.01.

Next, we examined whether systemic administration of RvD1 treatment rescued the lupus phenotype in MRL*/lpr* mice. When compared to control group, the RvD1 treatment significantly reduced the size of spleens and lymph nodes (**Figures 3A, B**) and decreased serum levels of IgG, anti-dsDNA (**Figures 3C, D**). Serum ANA level showed a tendency to decrease (**Figure 3E**). Renal impairments were also ameliorated in the RvD1 treated group, as shown by lower proteinuria (**Figure 3F**) and reduced glomerular enlargement and hypercellularity (**Figure 3G**). Furthermore, pro-inflammatory cytokines (TNF-α, IFN-γ and IL-6) and a Th17 cell-related cytokine (IL-17) were decreased, while Treg cell-related cytokines (IL-10 and TGF-β) continued to increase, thus indicating that RvD1 inhibits inflammatory responses effectively and also modulates the Treg/Th17 imbalance (**Figures 3H, I**).

### RvD1 Regulated the Differentiation of Treg and Th17 Cells

**Figure 4:**
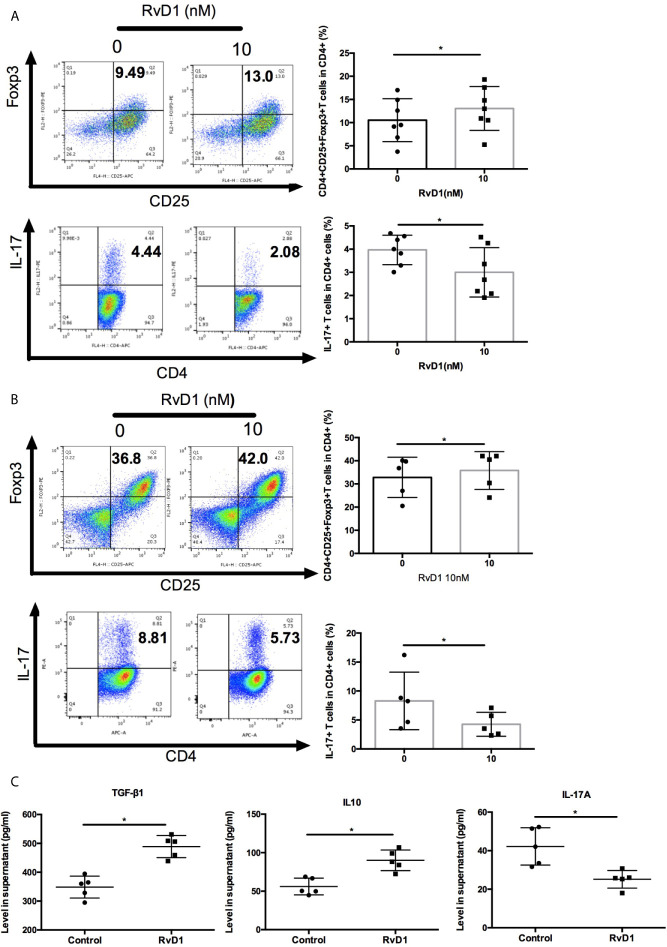
RvD1 modulates the proliferation and differentiation of Treg and Th17 in PBMCs and naïve CD4+ T cells in SLE patients. **(A)** The percentages of Treg and Th17 in human PBMCs with and without RvD1 treatment (n=7). **(B)** Flow cytometric analysis of Treg and Th17, which were differentiated from human naïve CD4+ T cells in the presence or absence of RvD1 (n=5). **(C)** The level of TGF-β, IL-10, and IL-17 in the culture supernatant were detected by ELISA (n=5). All data are mean ± SEM. *p < 0.05.

Next, we explore the effects of RvD1 on regulation Treg and Th17. Firstly, we cultured PBMCs from SLE patients with or without RvD1 and showed that RvD1 treatment increased the numbers of Treg, but suppressed Th17 (**Figure 4A**). We then investigated whether RvD1 influenced the differentiation of Treg and Th17. Naïve CD4+ T cells were purified from SLE patients and then cultured to differentiate into Treg or Th17 in the absence and presence of RvD1. We found that RvD1 treatment promoted Treg generation, but inhibited the differentiation of Th17 from naïve CD4+ T cells compared with control (**Figure 4B**). In addition, RvD1 increased the TGF-β and IL-10 but decreased IL-17 level in the culture supernatant (**Figure 4C**). These data suggest that RvD1 increases Treg and suppresses Th17 in SLE patients.

### RvD1 Increased Treg Differentiation and Inhibited Th17 Differentiation by Upregulating miR-30e-5p

**Figure 5:**
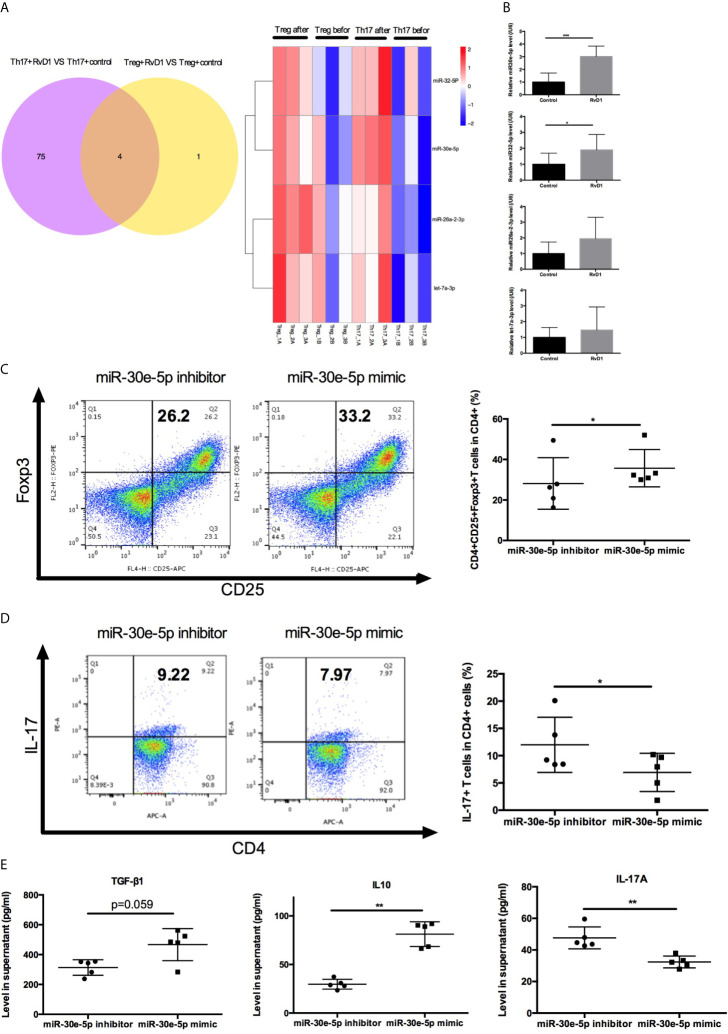
Effect of miR-30e-5p on the differentiation of naive CD4+ T cells from patients with SLE. **(A)** Venn diagram and heatmap analysis of miRNA expression profiles in Treg and Th17 differentiation from naive CD4+ T cells before and after RvD1 treatment. **(B)** The expression of miR-30e-5p, miR-32-5p, miR-26a-2-3p and let-7a-3p were verified by qPCR in independent SLE patients (n=10). **(C, D)** Representative flow cytometric plots indicated the percentage of Treg and Th17 cells polarized from naive CD4+ T cells transfected with miR-30e-5p mimic or inhibitor. **(E)** The level of TGF-β1, IL-10, and IL-17 in supernatant were detected by ELISA. All data are mean ± SEM. n=5 per group. ***p < 0.001.

To investigate the detailed mechanisms of miRNAs targeting Treg/Th17 balance by RvD1 in SLE, the expression profile of miRNA was analyzed by UMI Small RNA Sequencing in naive CD4+ T cells treated with RvD1. Four miRNAs were verified to up-regulate Treg differentiation and down-regulate Th17 differentiation cooperatively (**Figure 5A**). MiR-30e-5p, miR-32-5p, miR-26a-2-3p and let-7a-3p were selected for further qPCR verification experimentation. The expression of miR-30e-5p was dramatically upregulated after RvD1 treatment (**Figure 5B**). Next, the effect of miR-30e-5p on naive CD4+ T cell differentiation was investigated. The results showed that miR-30e-5p increased the proportion of Treg while it decreased the proportion of Th17 (**Figures 5C, D**). Additionally, miR-30e-5p increased the level of TGF-β and IL-10 while decreasing IL-17 levels in the culture supernatant (**Figure 5E**).

## Discussion

Chronic inflammatory in autoimmune disease occurs as a result of metabolic dysregulation, which is due to either uncontrolled activity of pro-inflammatory responses or inefficient resolution of inflammation. Resolvins might reduce inflammation through the stimulation of a number of signaling pathways (18). RvD1 plays a role in resolving acute inflammation, and a number display evidence for RvD1 mediating functions that are conserved in chronic inflammatory conditions, including RA, Sjogren’s syndrome, Parkinson’s disease and type 1 diabetes mellitus (19–22). It should be noted that the lower plasma level of RvD1 is not universal in inflammation disease, where an exception has been reported in patients with inflammatory arthritis by Barden et al. (23). Actually, conditions characterized by persistent or unresolved inflammation, cancer, aspirin and fish oil are associated with altered metabolism and function of pro-resolving mediators (24–27), so we conducted strict screening when recruiting patients. Herein we demonstrate that RvD1 level was lower in active SLE patients compared with inactive patients or healthy controls, which were consistent with prospective studies (11). Furthermore, a negative correlation between RvD1 level and SLEDAI score was observed. In addition, for part of the patients whom receiving relevant clinical test, the dynamic change of RvD1 was associated with alterations in dsDNA antibody level in response to disease activity in lupus. The mechanism underlying the transition of RvD1 from inactive to active phase remains unclear, as does whether it participates in the autoantibody production and increase the risk of autoimmunity. It is also worth mentioning that the limitations of our study included unavailability of comprehensive clinical data and relatively small sample sizes.

Importantly, the findings of MRL/*lpr* mouse model examining the time course of RvD1 reduction have strengthened our observation of the SLE patients, providing a translational significance. Specifically, it suggests that the RvD1 boost in 8-week-old mice could reflect an attempt to fight inflammation at its initial stages, being a molecule of early intervention. Our data indeed indicated that RvD1 intervention successfully ameliorated disease phenotype and corrected immune abnormality. It is in line with its well-known anti-inflammatory properties, and particularly provides strong organ protection against lupus nephritis. In addition, a higher proportion of Treg and a lower proportion of Th17 in spleens and lymph nodes were detected after RvD1 treatment, which broaden our understanding of its target cells. Furthermore, we show the presence of IL-10 and TGF-β with enhanced suppressive capability, which is different from the accumulation in resolving exudates.

Breaking the Treg/Th17 balance in peripheral blood is suggested to contribute to the pathogenesis of SLE and pro-inflammatory response, especially in the active form of the disease (28). Accordingly, we cultured PBMCs from SLE patients with or without RvD1 and showed that RvD1 treatment increased the numbers of Treg, but suppressed Th17. Furthermore, we conducted the naive CD4+ T cell differentiation experiments and showed that RvD1 could promote Treg differentiation while inhibiting Th17 polarization. There are consistent with mouse studies of RvD1 treatment.

The elucidation of mechanism that RvD1 mediates Treg/Th17 imbalance could provide useful information for designing novel treatment strategies for managing lupus symptoms. In this respect we focused on miRNAs, which are emerging as key regulators through the inflammation resolution process governed by RvD1 (29, 30). We identified a novel target, miR-30e-5p, as a downstream microRNA mediating the restoration of RvD1 on Treg/Th17 imbalance in SLE patients. In previous studies, miR-30e-5p has been found as a promising biomarker in the diagnosis and clinical manifestation of several diseases, including SLE and Diabetes (31, 32). In addition, miR-30-5p has been reported as a tumor suppressor and potential therapeutic nanomedicine in cancer (33–35). Furthermore, miR-30e-5p could attenuate obesity and adipose tissue type 1 inflammation through down regulating Th1 cells (36). MiRNAs have different target genes in various diseases and exert diverse regulatory functions. The target genes of miR-30e-5p in response to RvD1 were not confirmed in this study and require further investigation to allow for the molecular mechanism of miR-30e-5p in the regulation of Treg/Th17 balance. Related researches showed that miR-30e-5p acts as an oncogene in the progression of NSCLC by influencing STAT3 signaling (37). Another study showed the regulatory relationship between miR-30e-5p and PTEN and its effect on alleviation of myocardial infarction (38). Coincidently, PTEN-regulated STAT3 signaling is necessary for proper Treg and Th17 differentiation and function.

In conclusion, in the present study we showed for the first time that RvD1 effectively ameliorates SLE progression through up-regulating Treg and down-regulating Th17 cells by miR-30e-5p.

## Data Availability Statement

The microRNA sequencing data has been uploaded to NCBI— http://www.ncbi.nlm.nih.gov/bioproject/705865 (BioProject ID: PRJNA705865).

## Ethics Statement

The studies involving human participants were reviewed and approved by Ethics Committee of The Affiliated Drum Tower Hospital, Medical School of Nanjing University. The patients/participants provided their written informed consent to participate in this study. The animal study was reviewed and approved by Committee of Experimental Animal Administration of The Affiliated Drum Tower Hospital, Medical School of Nanjing University.

## Author Contributions

TC and SD participated in study design, data collection, data analysis, data interpretation, and drafting the paper. SL, XL, and XT participated in patient recruitment, animal experiments, and data collection. LS supervised the whole research, designed the study, interpreted the data, and wrote the paper. All authors contributed to the article and approved the submitted version.

## Funding

This research was funded by National Key R&D Program of China (2020YFA0710800); Major International (Regional) Joint Research Project of China (81720108020); Fundamental Research Funds for the Central Universities (YG2005002); Key Program of National Natural Science Foundation of China (81930043); Jiangsu Provincial Key Research and Development (BE2020621).

## Conflict of Interest

The authors declare that the research was conducted in the absence of any commercial or financial relationships that could be construed as a potential conflict of interest.
