# Supplementary material for: Resolvin D1 Improves the Treg/Th17 Imbalance in Systemic Lupus Erythematosus Through miR-30e-5p
Source: Front Immunol. 2021 May 19;12:668760. doi: 10.3389/fimmu.2021.668760 (PMC8171186; doi:10.3389/fimmu.2021.668760)
Supplement: Supplementary file 1 [file DataSheet_1.pdf]

Supplementary Material

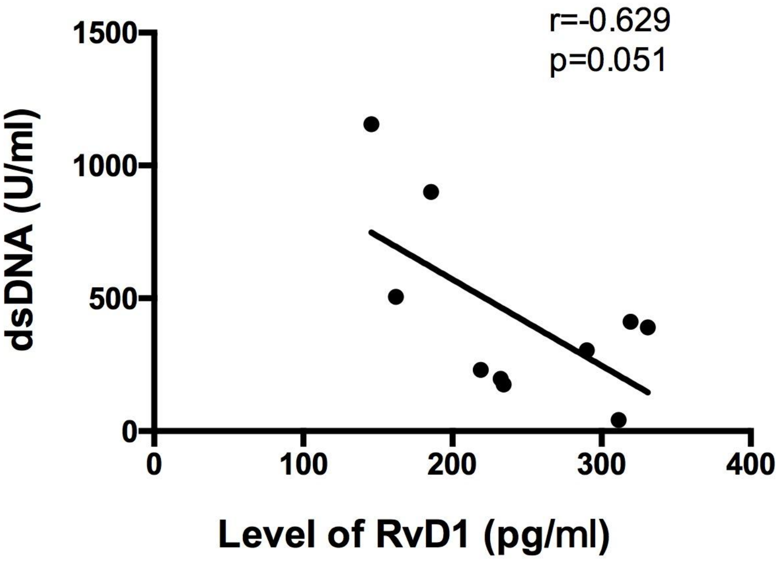

Figure S1. The dsDNA antibody level had a negative correlation trend with RvD1 level (r=-0.629, p=0.051). All data are mean ± SEM. n=10, $^{**}p<0.01$.
