# Supplementary material for: Resolvin D1 Improves the Treg/Th17 Imbalance in Systemic Lupus Erythematosus Through miR-30e-5p
Source: Front Immunol. 2021 May 19;12:668760. doi: 10.3389/fimmu.2021.668760 (PMC8171186; doi:10.3389/fimmu.2021.668760)
Supplement: Supplementary file 2 [file Table_1.docx]

Supplementary Material

**Table S1.** Baseline clinical characteristics and medications for the SLE patients

| **Patient**  **Age/sex** | **Disease duration**  **(Months)** | **Baseline**  **SLEDAI** | **Clinical manifestations** | **Medication** |
| --- | --- | --- | --- | --- |
| 27/female | 12 | 1 | C, ANA+, anti-dsDNA+ | Pred, HCQ |
| 27/female | 6 | 5 | A, C, H, ANA+, anti-dsDNA+ | Pred, HCQ |
| 36/female | 24 | 5 | F, LN, H, ANA+, anti-dsDNA+ | Pred, CYC, Aza, HCQ |
| 37/male | 10 | 8 | C, R, F, LN, H, ANA+, anti-dsDNA+ | Pred, MMF, HCQ |
| 34/female | 1 | 2 | C, ANA+, anti-dsDNA+ | Pred, HCQ |
| 40/male | 18 | 6 | LN, P, H, ANA+, anti-dsDNA+ | Pred, CYC, HCQ |
| 44/male | 3 | 4 | A, H, ANA+, anti-dsDNA+ | Pred, HCQ |
| 35/female | 5 | 4 | C, R, H, ANA+ | Pred, HCQ |
| 39/female | 1 | 17 | A, LN, F, C, H, ANA+, anti-dsDNA+ | Pred, HCQ |
| 25/female | 6 | 26 | R, M, LN, H, ANA+, anti-dsDNA+ | Pred, LEF, HCQ |
| 44/female | 24 | 12 | V, LN, H, ANA+, anti-dsDNA+ | Pred, CYC, MMF, HCQ |
| 36/female | 7 | 16 | LN, NP-SLE, H, ANA+, anti-dsDNA+ | Pred, CYC, HCQ |
| 26/male | 2 | 13 | V, R, F, C, H, ANA+, anti-dsDNA+ | Pred, HCQ |
| 27/female | 2 | 13 | LN, R, P, F, H, ANA+, anti-dsDNA+ | Pred, HCQ |
| 30/female | 0.5 | 16 | LN, P, F, C, H, ANA+, anti-dsDNA+ | Pred, HCQ |
| 33/female | 10 | 12 | LN, R, C, H, ANA+, anti-dsDNA+ | Pred, MMF, Aza, HCQ |
| 21/female | 1 | 7 | LN, R, C, H, ANA+, anti-dsDNA+ | Pred, HCQ |
| 26/female | 3 | 2 | R, H, ANA+ | Pred, HCQ |
| 32/female | 36 | 16 | LN, P, C, H, ANA+, anti-dsDNA+ | Pred, CYC, HCQ |
| 30/female | 144 | 4 | LN, H, ANA+, anti-dsDNA+ | Pred, HCQ, Tac |
| 21/female | 72 | 20 | V, LN, Alo, H, ANA+, anti-dsDNA+ | Pred, MMF, HCQ, Tac |
| 31/female | 120 | 10 | A, LN, R, H, ANA+, anti-dsDNA+ | Pred, MMF, HCQ |
| 33/female | 96 | 8 | A, LN, ANA+, anti-dsDNA+ | Pred, HCQ |
| 28/female | 1 | 15 | LN, R, F, C, H, ANA+, anti-dsDNA+ | Pred, HCQ |
| 27/female | 36 | 12 | LN, R, P, H, ANA+, anti-dsDNA+ | Pred, HCQ |
| 30/female | 24 | 5 | A, C, H, ANA+, anti-dsDNA+ | Pred, HCQ,  CyA |
| 29/female | 120 | 8 | LN, H, ANA+, anti-dsDNA+ | Pred, CYC, HCQ |
| 23/female | 96 | 11 | LN, P, F, H, ANA+ | Pred |
| 26/female | 144 | 12 | LN, V, H, ANA+, anti-dsDNA+ | Pred, HCQ |
| 48/female | 108 | 1 | C, ANA+ | Pred |
| 18/female | 0.5 | 4 | R, F, C, ANA+, anti-dsDNA+ | Pred, HCQ |
| 40/female | 1 | 8 | LN, P, C, H, ANA+ | Pred, HCQ |
| 35/female | 8 | 4 | A, H, ANA+, anti-dsDNA+ | Pred, HCQ, MTX |
| 40/female | 12 | 2 | R, ANA+, anti-dsDNA+ | Pred, HCQ |
| 38/female | 24 | 4 | LN, H, ANA+, anti-dsDNA+ | Pred, MMF, HCQ |

### Alo, alopecia; A, arthralgia; Aza, azathioprine; ANA, antinuclear antibody; anti-dsDNA, anti double strand DNA antibody; C, cytopenia; [CyA, Cyclosporine A](http://www.baidu.com/link?url=2BwkUqtX0osqHTbm3VuqUuN7QSCbvqmg2iVO7pJ0ix8BuOSvs_hyoYVonGB6kTqc3rZs-f5vBWrVtihnjYH64K); CYC, cyclophosphamide; F, febrile; H, hypocomplementemia; HCQ, hydroxychloroquine; LEF, leflunomide; LN, lupus nephritis; MMF, mycophenolate mofetil; MTX, Methotrexate; M, myositis; NP-SLE, neuropsychiatric lupus; P, polyserositis; Pred, prednisone; R, rash; Tac, Tacrolimus; V, vasculitis;
